# Impact of a structured interview on beta-lactam reaction documentation quality

**DOI:** 10.1017/ash.2021.172

**Published:** 2021-07-12

**Authors:** Cynthia T. Nguyen, Randall W. Knoebel, Jennifer Pisano, Kenneth Pursell, Natasha N. Pettit

**Affiliations:** 1 Department of Pharmacy, University of Chicago Medicine, Chicago, Illinois; 2 Department of Medicine, Section of Infectious Diseases and Global Health, University of Chicago Medicine, Chicago, Illinois

## Abstract

Incomplete documentation of β-lactam reactions often leads to inappropriate antibiotic prescribing. The objective of this study was to evaluate the impact of a structured interview on the quality of β-lactam reaction documentation. After 203 interviews, documentation of the core components of a β-lactam reaction improved (48% vs 1%; *P* < .001).

Beta-lactam antibiotics are often first-line therapy for the prevention and treatment of infection. Unfortunately, self-reported β-lactam allergies are common: ∼10% of people in the United States have a documented β-lactam allergy.^1^ These self-reported β-lactam allergies have downstream consequences, including receipt of inappropriate antibiotic therapy and poor clinical outcomes.^2,3^ Incomplete documentation to describe the reaction contributes to the prescribing of less effective and more toxic antibiotics, such as fluoroquinolones and aminoglycosides.^4,5^ Improving the quality of β-lactam reaction documentation can be a powerful tool for stewarding resources, particularly at centers without allergy consultation services and/or the ability to perform penicillin skin testing.

Structured interviews have demonstrated the ability to improve antibiotic use among patients with β-lactam allergies even in the absence of penicillin skin testing.^6–9^ With the appropriate information, the reaction type can be classified and the risk of cross reactivity to other β-lactam antibiotics can be determined. Complete documentation in the electronic health record (EHR) can provide important information regarding the ability to challenge with the same or alternative β-lactam antibiotic. In November 2018, we developed a questionnaire to guide β-lactam reaction interviews in the inpatient setting. In this study, we evaluated the impact of structured interviews on the quality of β-lactam reaction documentation.

## Methods

### Study design

This single-center, prospective, quasi-experimental study was conducted among inpatients with a β-lactam allergy label. Hospitalized adult patients with a β-lactam allergy label who underwent an interview using the questionnaire between November 1, 2018, to November 31, 2019, were included. The quality of β-lactam reaction documentation was evaluated based on 3 core components: time of the reaction, timing in relation to initiation of the β-lactam course, and a description of the reaction. Documentation of interventions received in response to the reaction and tolerance of other β-lactams were also evaluated. Secondary outcomes included the number of β-lactam allergy labels removed (delabeled) and antibiotic use 1 year before versus 1 year after the interview among the included patients. Antibiotic use was assessed using a continuous measure of days of therapy (DOT) per inpatient day and a nominal measure of receipt or absence of antibiotic doses. Antibiotic DOT and inpatient days were obtained using our hospital’s charge master.

### Intervention

In November 2018, members of the Antimicrobial Stewardship Program (ASP), Section of Infectious Diseases and Global Health, and Department of Allergy and Immunology developed a questionnaire to guide β-lactam reaction interviews (Supplementary Material online). The questionnaire was available on the internal ASP website and was not embedded into the EHR. Patients with a β-lactam allergy label were identified for potential interview using a report in the EHR. Patients were randomly selected from this list by pharmacists, pharmacy residents, and pharmacy students during their ASP rotation experience (∼3 interviews per week). All patients with a beta-lactam allergy label were eligible to be interviewed, regardless of the need for antibiotics during the index admission, but patients receiving ciprofloxacin and aztreonam were prioritized. After the interview, pharmacists updated the documentation in the allergy field in the patient’s medical record. If a reaction was eligible for deletion (eg, family history, entered in error, etc), the patient was provided verbal education and asked whether the allergy could be removed from the chart. Throughout the study period, inpatient allergy consultation and penicillin skin testing were not available.

### Statistics

We compared paired data among the same patients before and after the intervention. Nominal variables were compared using the McNemar test. Ordinal or nonparametric data were compared using the Wilcoxon signed-rank test. Comparisons were based on valid data for each mediation type listed above. Tests of significance were 2-tailed and *P* ≤ .05 was considered statistically significant. Data were analyzed using SPSS Statistics version 26 predictive analytics software (IBM, Armonk, NY).

## Results

During the study period, 203 adult patients with a β-lactam allergy label underwent a structured interview. The median time to complete an interview was 10 minutes (IQR, 5–15). Prior to the interview, the most recent documentation was completed by a nurse (47%), physician (23%), pharmacist (15%), medical assistant (12%), or other healthcare worker (3%). The median age of the study population was 62 years old (IQR, 47–70), and 93% of these patients were female (93%). Patients reported reactions to a penicillin (78%) and/or cephalosporin (29%). In total, 26 patients (13%) reported having a reaction to multiple β-lactam classes.

As a result of the interviews, 14 (7%) β-lactam allergy labels were removed from patient records. None of these patients had their allergy relabeled in the year after the interviews. Of the remaining patients, documentation of all 3 core components of the β-lactam reaction significantly improved after the interviews (48% vs 1%; *P* < .001). Upon interview, most patients were able to recall the reaction (94%) and how long ago it had occurred (92%), but only half of patients could recall the timing of the reaction in relation to the initiation of the medication (Table 1).

**Table 1.** Beta-Lactam Reaction Documentation Before and After the Interview

Reaction Documentation	Pre-interview (N = 203), No. (%)	Post-interview (N = 203), No. (%)	*P* Value
Allergy label deleted after the interview	NA	14 (7)	…
**Documented components of reaction history**	(n = 203)	(n = 189)	
All 3 core components	3 (1)	90 (48)	<.001
Description of reaction	162 (80)	178 (94)	<.001
Time of reaction	13 (6)	174 (92)	<.001
Timing of reaction in relation to therapy initiation	5 (2)	96 (51)	<.001
Interventions used to respond to reaction	5 (2)	74 (39)	<.001
Tolerance of other β-lactams	29 (14)	98 (52)	<.001
**Classification**	n = 203	n = 189	.004
Severe, IgE mediated	77 (38)	82 (43)	
Severe, non–IgE mediated	5 (2)	4 (2)	
Mild to moderate	61 (30)	71 (38)	
Intolerance	17 (8)	19 (10)	
Unknown or no description	41 (20)	11 (6)	
No allergy (eg, family history)	2 (1)	2 (1)	

Non–β-lactam gram-negative antibiotic use was lower in the postintervention period (63.2 vs 28.9 DOT per inpatient day). Individually, aztreonam (17 vs 6.8 DOT per inpatient day), aminoglycoside (6.8 vs 1.7 DOT per inpatient day), and fluoroquinolone (39.4 vs 20.3 DOT per inpatient day) use were also lower in the postintervention period. After the interviews, patients were less likely to receive non–β-lactam gram-negative antibiotics in the following year (67% vs 43%; *P* < .001).

## Discussion

We found an improvement in the quality of β-lactam reaction documentation after implementing structured interviews. A questionnaire can ensure that important components of the history are not omitted, and it allows the intervention to be performed by a variety of healthcare professionals (eg, students or technicians). This intervention can be particularly useful in resource-limited settings when inpatient allergy consultation or penicillin skin testing is not available. In a national US survey including 121 respondents, fewer than half of hospitals had access to inpatient allergy specialist consultations (44%) and inpatient penicillin skin testing (39%).^10^

Although many studies have evaluated the impact of β-lactam allergy interviews, our study is unique in several ways. While several studies have reported rates (34%–93%) of patients requiring any change in allergy documentation,^6–8^ our study quantifies the specific components of the history and better describes the quality of documentation. Based solely on an interview, we were able to delabel 7% of patients. In a similar study of 175 inpatients, only 1% of labels were delabeled.^8^ We also collected antibiotic use data beyond the index admission among the same patients, whereas previous studies have focused on antibiotic use during the admission in which the reaction history was obtained compared to a historic control group.^7–9^

Our study has several limitations. As a single-center study, similar results may not be observed at centers that provide guidance or have programs to optimize allergy documentation. We are also unable to attribute the observed changes in antibiotic use directly to the intervention. For example, evaluating antibiotic use in the same patients before and after the intervention introduces survivor bias, and antibiotic use would be affected by antibiotic discontinuation (if the patient no longer needs antibiotics).

Our study demonstrates important improvements in the quality of β-lactam reaction documentation as a result of a structured interview. These improvements may be linked to antibiotic prescribing and have the potential to translate to improved patient outcomes.

## Supplementary material

For supplementary material accompanying this paper visit https://doi.org/10.1017/ash.2021.172. click here to view supplementary material
