# Supplementary material for: Impact of a structured interview on beta-lactam reaction documentation quality
Source: Antimicrob Steward Healthc Epidemiol. 2021 Jul 12;1(1):e10. doi: 10.1017/ash.2021.172 (PMC9495407; doi:10.1017/ash.2021.172)
Supplement: Supplementary file 1 [file S2732494X21001728sup001.pdf]

Supplement: Comprehensive Beta-Lactam Reaction Interview Tool

Antimicrobial Stewardship Program | Allergy and Immunology | University of Chicago Medicine

Patient Name: ________________________________MRN: ____________________ Approx time (min) to complete: ________

Historian (check all that apply):

- ☐ Patient
- ☐ Parent/ Guardian
- ☐ Spouse/ Family
- ☐ Pharmacy
- ☐ Chart review

1. What beta lactam antibiotic(s) are you allergic to?

- ☐ Penicillin (indicate if PO, IV, or IM)
- ☐ Amoxicillin (Amoxil)
- ☐ Amoxicillin/clavulanate (Augmentin)
- ☐ Piperacillin/tazobactam (Zosyn)
- ☐ Cefazolin (Ancef)
- ☐ Cephalexin (Keflex)
- ☐ Cefdinir (Omnicef)
- ☐ Ceftriaxone (Rocephin)
- ☐ Cefepime (Maxipime)
- ☐ Other: ___________________

2. Why were you taking this antibiotic?

- ☐ Pneumonia (infection of the lungs)
- ☐ Sinus or upper respiratory tract infection
- ☐ Skin and soft tissue infection (i.e. cellulitis)
- ☐ Urinary tract infection (UTI)
- ☐ Unknown
- ☐ Other: ___________________

3. When did you last receive this antibiotic?

- ☐ Approx year: __________
- ☐ < 5 years ago
- ☐ 5-10 years ago
- ☐ >10 years ago

4. How long after receiving the antibiotic did you experience this reaction?

- ☐ After one dose (minutes – hours)
- ☐ Unknown
- ☐ After 2-5 days
- ☐ Other:_________________
- ☐ After >5 days

5. What signs or symptoms did you experience when you received the antibiotic?

IgE Mediated	Non-IgE Mediated	Mild	Intolerance
☐ Anaphylaxis	☐ Skin sloughing/ peeling (skin falling off)	☐ Rash (not hives, e.g. maculopapular)	☐ Nausea
☐ Difficulty breathing/ shortness of breath	☐ Lesions in mucosal areas (mouth, eyes, genitalia)	☐ Dizziness	☐ Vomiting
☐ Throat, tongue or lip swelling	☐ Anemia (destruction of red blood cells)	☐ Itching (no rash)	☐ Diarrhea
☐ Hives (see below)	☐ Hepatitis (liver dysfunction)		☐ Headache
	☐ Nephrotoxicity (kidney injury)		☐ Hallucinations
			☐ Sensitivity to light/sun
			☐ Muscle aches/ cramps

☐ Unknown ☐ Other: ____

6. How long did symptoms last? < 24 hours? 1-3 days? > 4 days?

- ☐ <24 hours
- ☐ 1-3 days
- ☐ >4 days

7. If you were outside the hospital, did you go to the hospital or emergency room for this reaction?

- ☐ Yes
- ☐ No
- ☐ Reaction occurred while in the hospital or emergency room

8. What interventions did you receive for this reaction?

- ☐ Intubation/ breathing tube
- ☐ EpiPen, epinephrine
- ☐ Diphenhydramine (Benadryl), steroids, antihistamines
- ☐ Self-resolved (none)
- ☐ Unknown

9. Have you ever had penicillin skin testing?

- ☐ Yes
- ☐ No
- ☐ Unknown
- ☐

If yes, when did testing occur? ______________

What was the result? ☐ Positive ☐ Negative ☐ Unknown

10. What beta-lactams have you tolerated after the allergic reaction?

- ☐ Penicillin (indicate if PO, IV, or IM)
- ☐ Amoxicillin (Amoxil)
- ☐ Amoxicillin/clavulanate (Augmentin)
- ☐ Piperacillin/tazobactam (Zosyn)
- ☐ Cefazolin (Ancef)
- ☐ Cephalexin (Keflex)
- ☐ Cefdinir (Omnicef)
- ☐ Ceftriaxone (Rocephin)
- ☐ Cefepime (Maxipime)
- ☐ Other: ___________________

11. If the patient states a dermatologic reaction, confirm the classification using images below.

Hives (itchy, raised welts, red with pale center)

[example images of hives]

Maculopapular rash (itchy, flat or raised)

[example images of maculopapular rash]

Please update Allergies field in Epic. Use the ".PENICILLINALLERGY" dot phrase to ensure documentation of all the appropriate information.

References: Shenoy ES et al. JAMA. 2019;321(2):188-199; Blumenthal K et al. J Allergy Clin Immunol Pract. 2014 ; 2(4): 407–413; Gruchalla RS et al. New Engl J Med. 354;6:601-609; Har D et al. Immunol Allergy Clin N Am 37 (2017) 643–662; Legendre DP et al. Clin Infect Dis. 2014;58:1140-1148; Limsuwan T, Demoly P. Med Clin North Am 2010;94(4): 691–710.
